# Gait Velocity Is an Indicator of Cognitive Performance in Healthy Middle-Aged Adults

**DOI:** 10.1371/journal.pone.0103211

**Published:** 2014-08-04

**Authors:** Artin Jabourian, Sylvie Lancrenon, Catherine Delva, Alain Perreve-Genet, Jean-Pierre Lablanchy, Maritza Jabourian

**Affiliations:** 1 Laboratory of Clinical Neurophysiology, Paris, France; 2 SYLIA-STAT, Bourg la Reine, France; 3 Center of Cardiology, Paris, France; 4 Center of Psychiatry, Paris, France; University Medical Center Groningen UMCG, Netherlands

## Abstract

Psychomotor retardation, especially motor and cognitive slowing down, has been described many times in the elderly but to our knowledge, has never been examined in healthy middle-aged adults. The present study explores whether walking time may provide an early signal of cognitive performance, using 266 healthy adults ([18–65] years old, mean age: 45.7±12.9 years) who were also subdivided in 2 groups: under or over 50. Walking time (50 meters) and cognitive performances (mini-mental state examination, Benton Visual Retention Test and Rey Complex Figure) were assessed; total psychometric score was the sum of individual test scores. Analyses were controlled for age, gender, education level, height and weight. The mean psychometric scores were within the normal range. A substantial proportion of subjects exhibited low performance in some aspects of visuospatial memory, particularly in the older subset. In the total population, walking time was negatively correlated with all cognitive tests, particularly to total psychometric score (R = −0.817, p<0.0001); the unique contribution of walking time on all cognitive scores was very high (delta R-squared = 0.496). In the older subset, performances on walk and cognition were lower than in the younger subset. Total psychometric score showed the strongest correlation with walking time in the older subset (R = −0.867; p<0.001). In all subsets, walking time was the main explanatory variable of the total psychometric score (delta R-squared: ≤ 49 = 0.361; ≥50 = 0.613). These findings indicate that i) a significant proportion of adults without cognitive complaints exhibit low cognitive performance including visuospatial memory and longer walking time, ii) cognitive functioning is strongly correlated to walking time in healthy middle-aged adults, iii) gait velocity (GV) could be an indicator of cognitive performance in some important cognitive domains. These results warrant further investigation because such data may represent a marker for the detection of middle-aged adults who are at risk for further cognitive decline.

## Introduction

Psychomotor retardation, especially cognitive and motor slowing down has been described many times in the elderly and or in patients but, to our knowledge, never in healthy adults. Obvious most of the time, it might be insidious thereby frequently overlooked. The detection and quantification of psychomotor retardation are based on dual but separate measurement of physical and mental performances. Gait velocity (GV) testing is simple and easy to administer. In contrast, the quantification of cognitive performance needs a detailed and time-consuming neuropsychological assessment which is rarely performed at patient's bedside. Therefore availability of easy and quick tools would help clinicians to simply evaluate the cognitive state of their patients.

The neuropsychiatric assessment of more than 3000 patients from our clinical practice (suffering from various conditions such as falls, fainting or cardiac arrhythmias) led us to develop a simple methodology to quantify cognitive performance. Our methodology showed, probably for the first time, a relationship between gait and cognition in these patients [1], [2]. Moreover, in patients with cardiac arrhythmias, an unexpected increase of normal or subnormal GV and cognitive (mainly visuospatial) performance was detected after implantation of a pacemaker, regardless of the age of patients, retrospectively revealing an insidious subclinical psychomotor retardation [3].

Later studies have shown that GV is related to cognitive status [4], demonstrating that even routine walking depends on complex cognitive processes [5]. Quantitative gait measures can predict the future risk of cognitive decline and dementia in initially non-demented older adults, can discriminate normal individuals from those with mild cognitive impairment (MCI) [6] and can even distinguish between amnestic and non-amnestic forms of MCI [7]. The speed of the GV decline in normal elderly individuals may predict the onset of MCI [8], and GV in MCI patients over 65 years of age may be predictive of further risk of dementia [9]. While currently restricted to populations of patients or to healthy elderly individuals, the detection of poor cognitive performance should ideally be made much earlier in life, preferably in a healthy population without cognitive complaints [10], [11]. When detection occurs earlier, the probability of success for future interventions is improved [12]. Our previous studies suggested that an unrecognized decline in cognition and gait is conceivable in apparently healthy adults [1], [2], [3]. As both gait and cognition depend upon brain function, their association described many times in the elderly can also be expected in adults. Overall, the data described above motivated the present research.

Our study therefore assessed 1) whether poor performances on walking time and cognitive tests exist and can be detected in healthy middle-aged adult volunteers, 2) whether walking time is related to cognitive functioning in this population, in particular to visuospatial abilities given that a decline is commonly reported in normal aging and in preclinical stages of dementia [13], and 3) whether the strength of this relationship was strong enough to consider GV as an indicator of cognitive performance.

## Materials and Methods

This research was completed in accordance with the Helsinki Declaration. The study was observational and naturalistic, did not involve any intervention or medicinal product administration and was performed on healthy subjects. Gait and psychological tests were analyzed. According to the French Public Health regulations and definition of biomedical research, Article L1121-1 Code de la Santé Publique, this study did not require submission for information or approval by an ethics committee. All participants gave their written informed consent prior to participating in the study. They were informed that the results obtained could be published but that their personal data would remain anonymous.

### Study population

Four hundred subjects aged between 18 and 65 years old were recruited at Laboratory of Clinical Physiology, part of a medical center in Paris. Subjects were orally informed about the existence of this study via the medical center staff. Volunteers were accompanying persons (74%), staff (nurses, technicians, students) (9%) and subjects coming from dental care and gynecologist departments of the center (17%). Subjects were screened to determine their eligibility for the study. The participants' health status was carefully assessed by 2 experienced medical doctors (a cardiologist and a neuropsychiatrist) and included a physical examination (neurological, locomotor and cardiovascular systems) and a mental examination (for identification of psychiatric disorders, neurologic disorders or mnesic complaints as these conditions can bias cognitive and gait measurements). Medical history and concomitant treatments were recorded. All subjects had a normal social life (evaluated by medical interview of the subject), most of them had an occupation and few were retired. Of 400 subjects, 266 adults were judged healthy and eligible to participate in the study. Excluded subjects presented physical/organic disorders (n = 84), including current or history of coronaropathy, respiratory diseases, hypertension with systolic blood pressure >140 mm Hg, migraine, epilepsy, rheumatism or arthritis, polyneuritis, diabetes mellitus, history of thyroid dysfunction or subjective complaints (fatigue or pain of inferior limbs). Thirty-six subjects were excluded because they presented a history of depressive/anxious disorders (DSM-IV-TR confirmed by the Beck Depression Inventory and HAM-Anxiety scale), other mental disorders or sleep disorders. Subjects who were taking medication that could affect gait or mental status were excluded (n = 14).

### Gait performance

Walking time evaluations were conducted independently of psychometric testing. Subjects were required to walk with their regular shoes on level ground for 50 meters (25 meters back and forth: distance long enough to include numerous walk cycles) in a well-lit corridor, 2 meters wide and ending in a wall. From an initial line and standing still, the given order was: “Walk as fast as you can, without running, touch the wall and come back.” This order comprised a psychomotor test consisting of two motor performances (walk and turn back) and five cognitive orders (walk, as fast as you can, no running [thus evaluating the inhibitory aspect of executive functions], touch the wall, come back). The walking time (in seconds) was measured with a stopwatch. The start, turning and end were included in the walking time. We used the same methodology as in our previous studies [1], [2].

### Psychometry

Three widely known validated tests that were rapid to administer (less than 1 hour) were used to explore different subdomains of cognition and particularly visuospatial abilities (deficits in visuospatial abilities have been commonly reported in preclinical stages of dementia [13], [14]).

The Rey Osterrieth Complex Figure Test (ROCFT) includes the Rey copy and Rey memory subtests, which explore visuospatial and executive functions. This test explores most cognitive functions except verbal functions [15], [16], [17]. The complex figure is composed of geometric elements without any apparent meaning but gives a lot of psycho-neurophysiological information to the examiner. The subject was first instructed to copy the complex figure (Rey copy). When he had finished, the template and the subject's copy were withdrawn. Three minutes after and without forewarning, the participant was asked to recall the complex figure (Rey memory). Cognitive processes regarding perception and analysis strategies, organizational and constructional approach when drawing the figure were explored with the ROCFT. The recall subtest (Rey memory) explored spatial and working memory. The ROCFT is used in children to assess learning and neuro-psycho-developmental problems and in adult or elderly, as a tool to measure cerebral function and its eventual disturbance [16]. The 18-point system proposed by Osterrieth and adapted by Lezak was used, with norms for the age range 18–65 years being: Rey copy score >32/36 and Rey memory score >22/36 for percentile 50. A score under these thresholds was considered as low performance. Scores below percentile 25 were clearly abnormal: <30/36 and <13/36 respectively for the copy and the memory subtests [17], [18].

The M form of the Benton Visual Retention Test (Benton test) explores visuospatial memory without graphic reproduction. Individuals had to recognize a previously presented geometric figure among 4 figures. The thresholds of normality for the Benton Visual Retention Test in adults (18–65 years old) ranged in six categories from the superior (14/15) to inferior (11/15) bounds. A score lower than 10/15 was considered as clearly abnormal [19]. In the present study, the scores were doubled to homogenize the Benton test score with the scores from other tests and the middle cut-off score of 24/30 was considered as the threshold of low performance.

Overall cognitive functioning was assessed using the mini-mental state examination (MMSE), which evaluates cognitive function in several domains including orientation, registration, attention, construction skills, memory and language, with more than 15 verbal items. The MMSE is well correlated with the verbal IQ of the Wechsler Adult Intelligence Scale (WAIS and WAIS-R) and provides a reasonable measure of overall cognitive functioning [20]. The threshold of normality for the MMSE total score was set at 26/30 to detect subtle impairments that were otherwise not detectable with the standard 24/30 cut-off score [21].

The total psychometric score (TS) is the sum of the four above-mentioned psychometric tests scores (Rey copy, Rey memory, Benton Visual Retention, MMSE), a TS score lower than 104 (sum of individual tests thresholds) was considered as a low performance, a TS <87 was considered as clearly abnormal. The TS (range: 0–132) was used as a composite index.

Height, weight and number of years of education (from the age of 6 years old) were recorded.

### Statistical analysis

Cronbach's alpha test was applied to check for internal consistency among the different cognitive tests. Psychometric scores and walking time were compared between two age subsets (volunteers ≤ 49 years old and volunteers ≥ 50 years old) using the Mann-Whitney U-test. Percentages of low performers were compared using a chi-square test. Relationships between quantitative variables were studied by the Pearson correlation coefficients.

Simple linear regressions with the cognitive tests scores as explained variables and walking time as explanatory variable were performed. Then, step-wise multiple linear regressions were performed with the cognitive tests scores as explained variables and walking time, age, gender, education level, height and weight as explanatory variables. Age and education level were forced into the model, as they are well known to be related to walking time and/or cognition.

To validate the results, another statistical approach was performed: a hierarchical multiple linear regression analysis. The cognitive tests scores were the explained variables. First, all confounders (age, gender, education level, height and weight) were forced into the model then walking time was added.

For each regression analysis, the unique contribution of the walking time was calculated.

For the total psychometric score only, these analyses were conducted on the whole population and also on each age subset.

Statistical analysis was performed using SAS^®^ software (version 8.2, SAS Institute Inc., Cary, NC). Type I error was set at 5%.

## Results

### Characteristics of the population, global and by age subsets

The mean age of the studied volunteers (n = 266) was 45.7±12.9 years; females constituted the majority of the study population (63.9%). The mean height was 166±9 cm, and mean weight was 69.2±14.7 kg. The educational level was 10.6±2.4 years from the age of 6 years old. Height, weight and education were comparable between younger and older subsets.

Clinical characteristics, the results of the psychometric tests and walking time are presented in Table 1. In the whole study population, the mean Rey copy, MMSE and Benton test scores were within or above the normal range. The frequency of subjects with poor performance in these tests was low (e.g., Rey copy: 20.7%; MMSE: 17.3%). Concerning the Rey memory test, the mean score (18.38) was below the usual normal score for this age range (set at 22 for percentile 50 and affected 71.4% of the subjects). Few subjects had a clear abnormal score (thresholds described in the methodology section): 13.5% (n = 36/266) for the TS, 12% for the Rey copy, 20.6% for Rey memory, 17.3% for the Benton test and 10% for the MMSE, most of them (86%) were in the older subset and none of the scores was dramatically low. When comparing the data across age subsets, all cognitive tests scores were lower in subjects 50 years of age and over than in younger subjects (Table 1). Low performances on the tests were significantly more frequent in the older subset than in the younger one, except for the Rey memory (p = 0.29). The internal consistency of psychometric test scores was good (Cronbach's alpha coefficient, 0.82).

**Table 1:** Age, walking time and cognitive test scores.

		Whole study	Subset 1:	Subset 2:	Comparison
		population	Subjects ≤ 49	subjects ≥ 50	between
		(n = 266)	years old (n = 137)	years old (n = 129)	subsets^(1)^
**Age**		45.7 ± 12.9	35.3 ± 9.1	56.8 ± 4.4	-
**Walking time**		31.8 ± 7.8	28.9 ± 6.0	34.9 ± 8.4	<0.0001
**Total**	**Score**	101.8 ± 16.3	106.7 ± 11.1	96.5 ± 19.1	<0.0001
**psychometric**	**% of low**	51.50%	40.10%	63.60%	0.0001
**score**	**performers (<104)**				
	**Score**	23.0 ± 4.9	24.8 ± 3.3	21.0 ± 5.5	<0.0001
**Benton Test**	**% of low**	44.00%	28.50%	60.50%	<0.0001
	**performers (<24)**				
	**Score**	33.0 ± 4.5	34.2 ± 2.4	31.8 ± 5.8	0.0054
**Rey copy Test**	**% of low**	20.70%	10.20%	31.80%	<0.0001
	**performers (<32)**				
	**Score**	18.4 ± 7.4	19.7 ± 6.6	16.9 ± 8.0	0.0026
**Rey memory**	**% of low**	71.40%	68.60%	74.40%	0.29
**Test**	**performers (<22)**				
	**Score**	27.4 ± 3.2	28.0 ± 2.3	26.8 ± 3.9	0.0171
**MMSE**	**% of low**	17.30%	8.80%	26.40%	0.0001
	**performers (<26)**				

Values are expressed as the mean ± standard deviation in the whole study population and in the two subsets of subjects by age range. Percentages correspond to the ratio of low performers for each item. P values denote highly significant differences between the two age subsets.

(1) Comparisons between subsets are performed by a Mann-Whitney *U*-test for the scores and walking time (non normal distributions) and by a chi-square test for the % of subjects with low scores.

Walking time was significantly longer in the older subset (p<0.0001).

### Relationship between cognitive tests and walking time in the global population

Significant correlations were found between walking time and height (R = −0.30; p<0.0001), weight (R = −0.13; p = 0.03), sex (R = −0.29; p<0.0001) and education (R = −0.20; p = 0.001).

Education and cognitive performance were differentially correlated according to the tests: MMSE R = 0.18 (p = 0.0032), Rey copy R = 0.20 (p = 0.001), Rey memory R = 0.23 (p = 0.0002), Benton test R = 0.24 (p<0.0001) and TS: R = 0.27 (p<0.0001).

Age was significantly correlated with walking time (R = 0.41; p<0.0001) and with all cognitive tests. The Benton test and TS were the most sensitive to age (R = −0.340 and −0.305, respectively; p<0.0001), and significant correlations for other tests were as follows: Rey copy R = −0.26 (p<0.0001), Rey memory R = −0.19 (p = 0.0015) and MMSE R = −0.20 (p = 0.0009).

**Figure 1:**
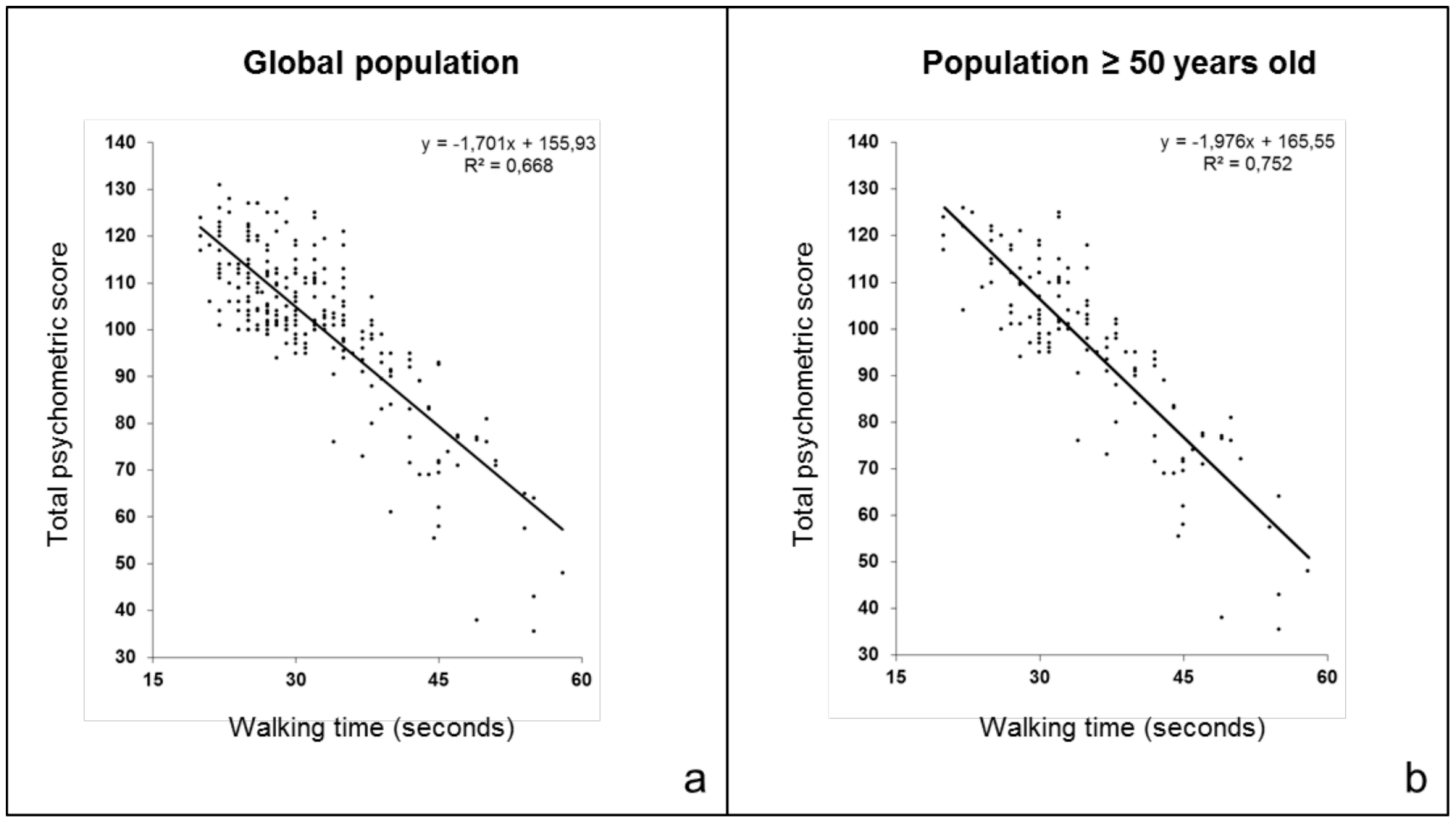
Total psychometric score and walking time. The relationship between the total psychometric score and walking time is visualized with a dot plot of the data and a linear regression of the total psychometric score and walking time in the whole study population (a) and in the subset of volunteers aged 50 years and over (b). The linear regression equations and R squared values are indicated in the figures.

**Figure 2:**
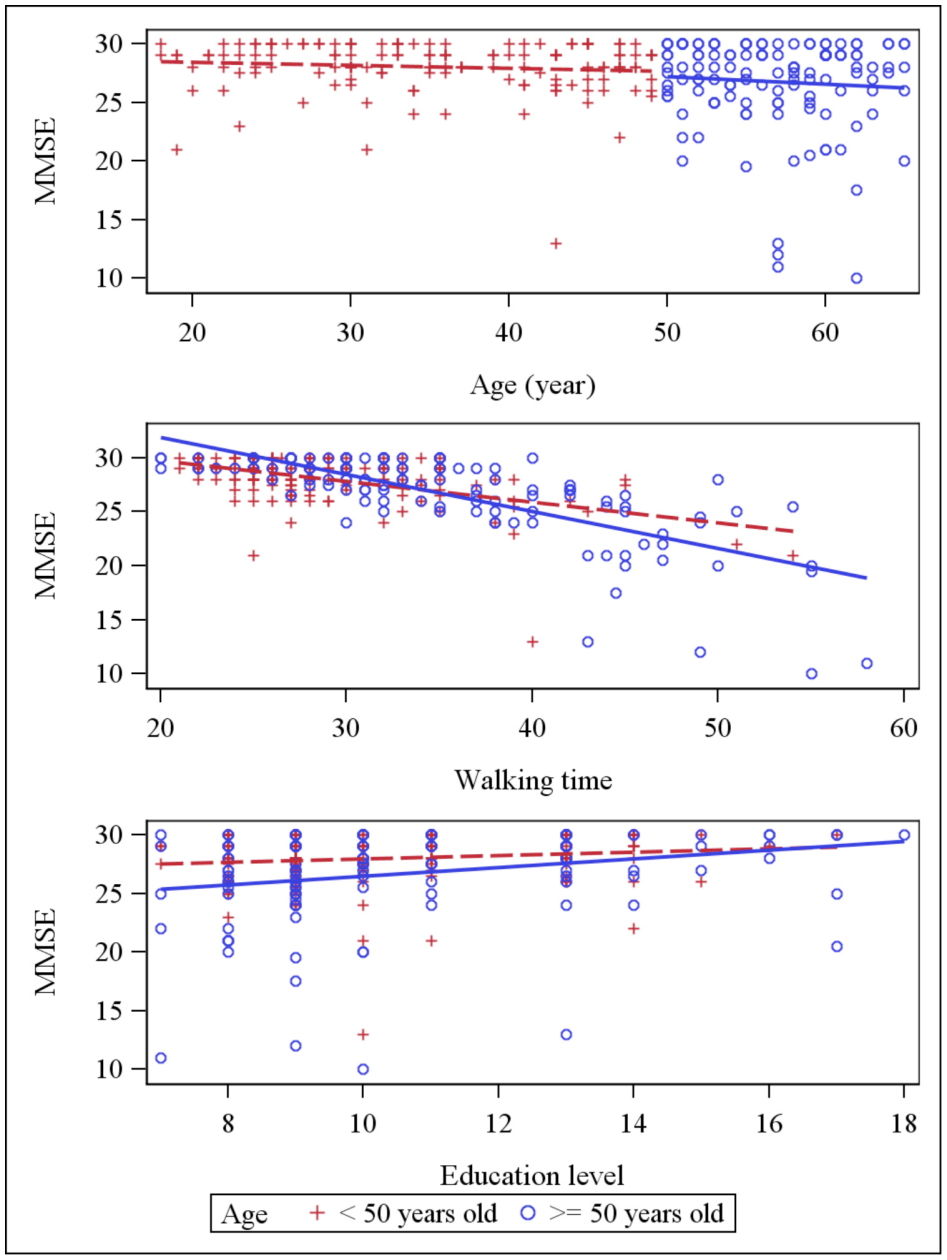
Scatter plot: MMSE score and explaining variables (age, education and walking time). Dot plots and linear regression for MMSE score against the main explaining variables: age, education and walking time. Subjects <50 years old are represented by red crosses, subjects ≥ 50 years old are represented by blue circles.

**Figure 3:**
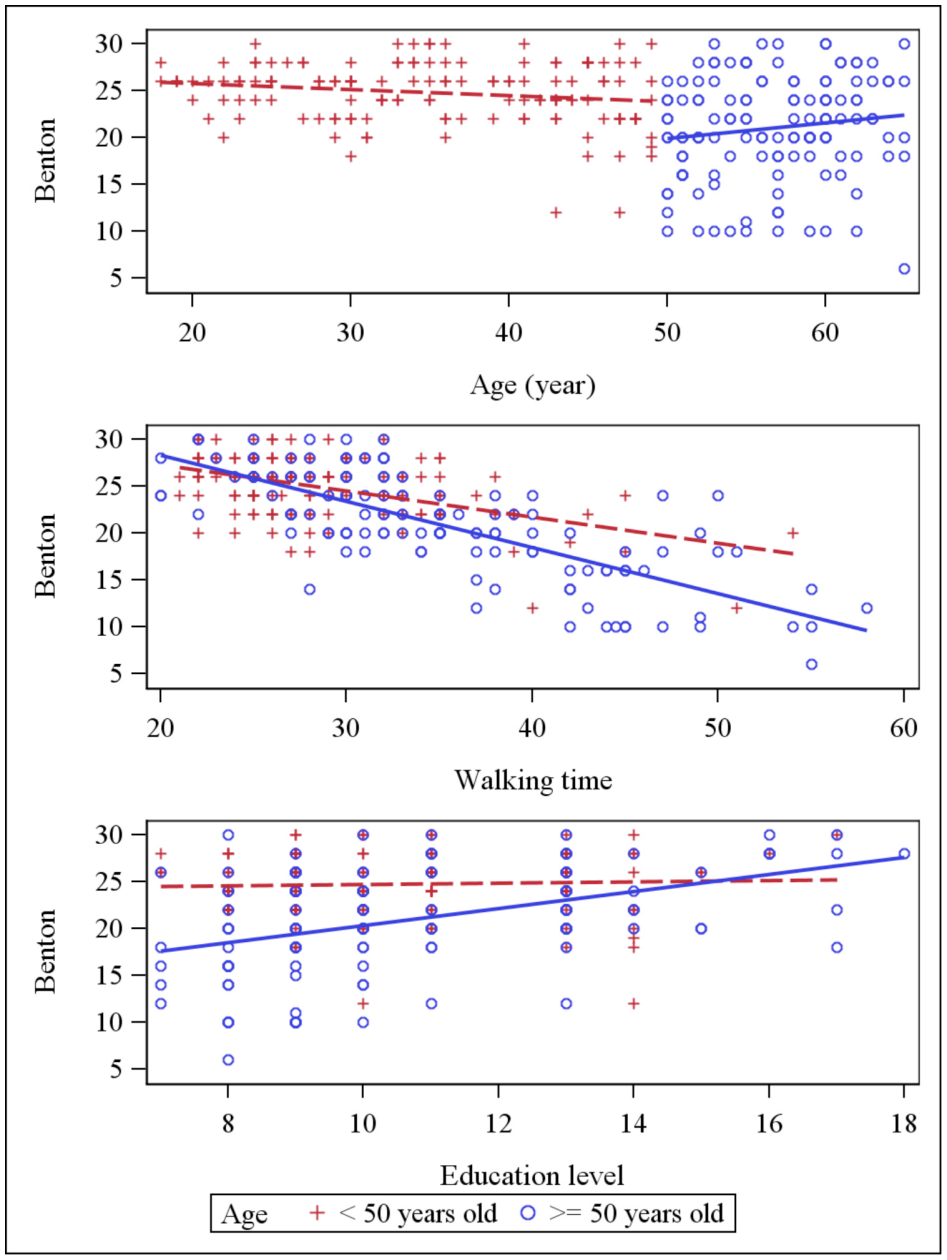
Scatter plot: Benton score and explaining variables (age, education and walking time). Dot plots and linear regression for Benton score against the main explaining variables: age, education and walking time. Subjects <50 years old are represented by red crosses, subjects ≥ 50 years old are represented by blue circles.

**Figure 4:**
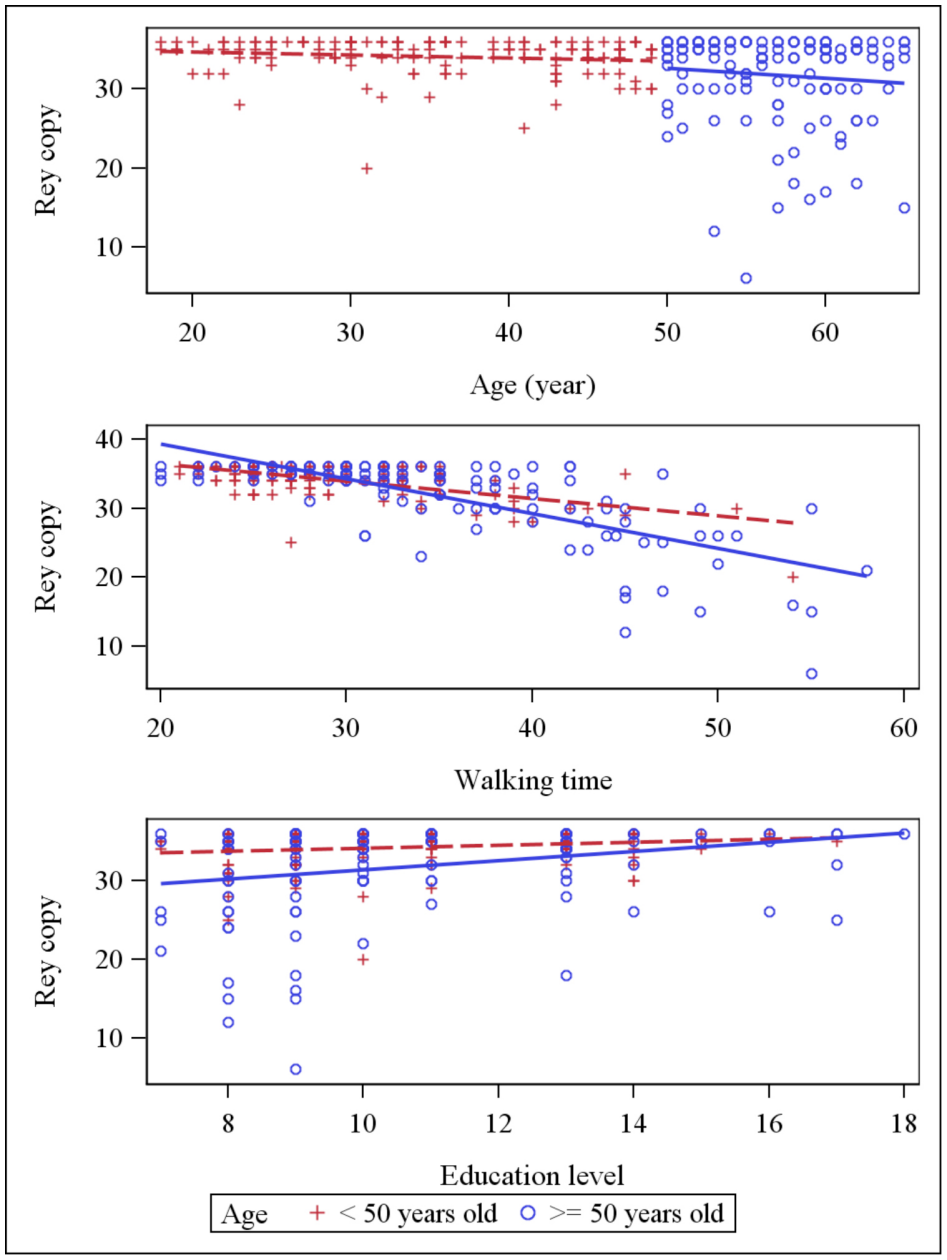
Scatter plot: Rey copy score and explaining variables (age, education and walking time). Dot plots and linear regression for Rey copy score against the main explaining variables: age, education and walking time. Subjects <50 years old are represented by red crosses, subjects ≥ 50 years old are represented by blue circles.

**Figure 5:**
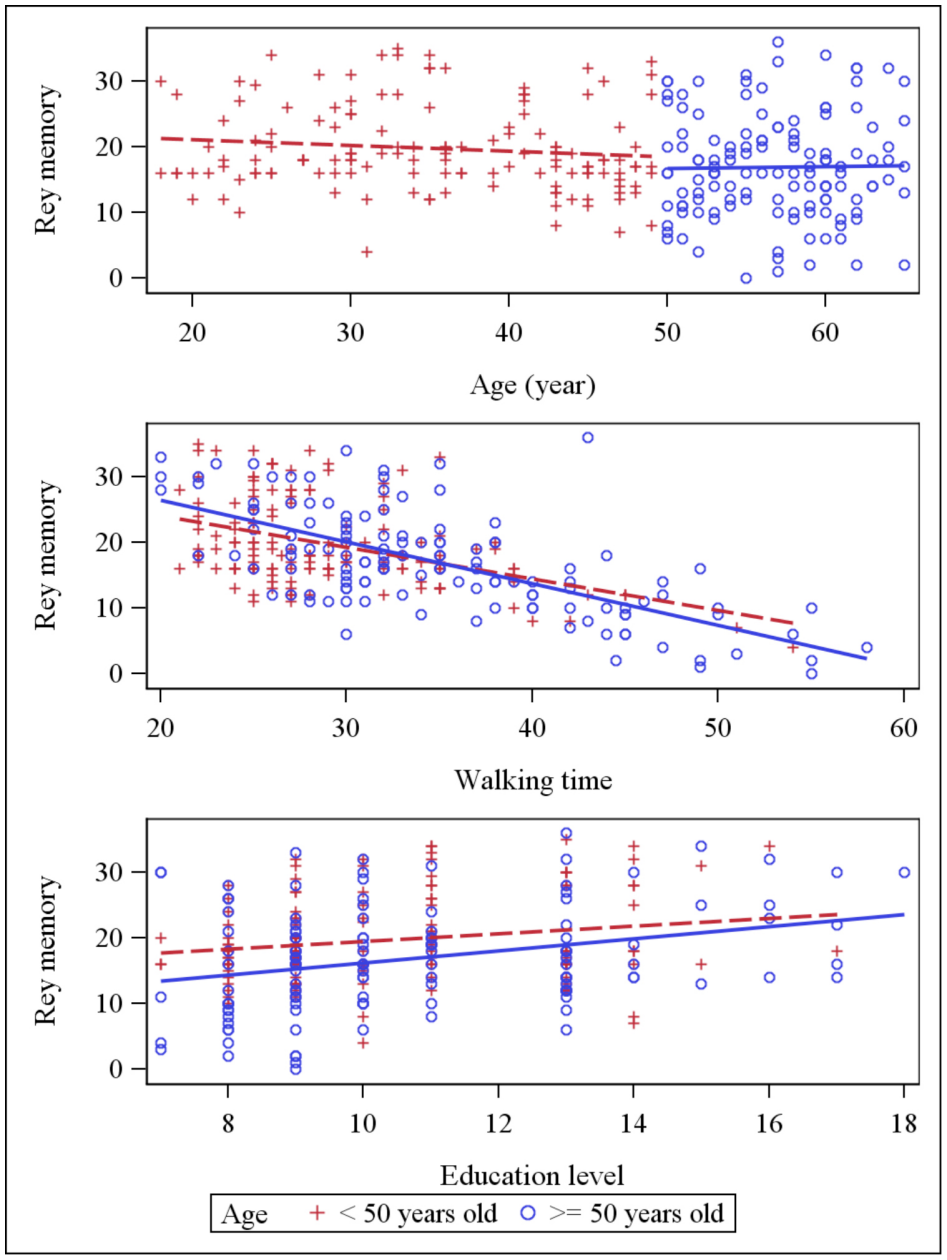
Scatter plot: Rey memory score and explaining variables (age, education and walking time). Dot plots and linear regression for Rey memory score against the main explaining variables: age, education and walking time. Subjects <50 years old are represented by red crosses, subjects ≥ 50 years old are represented by blue circles.

**Figure 6:**
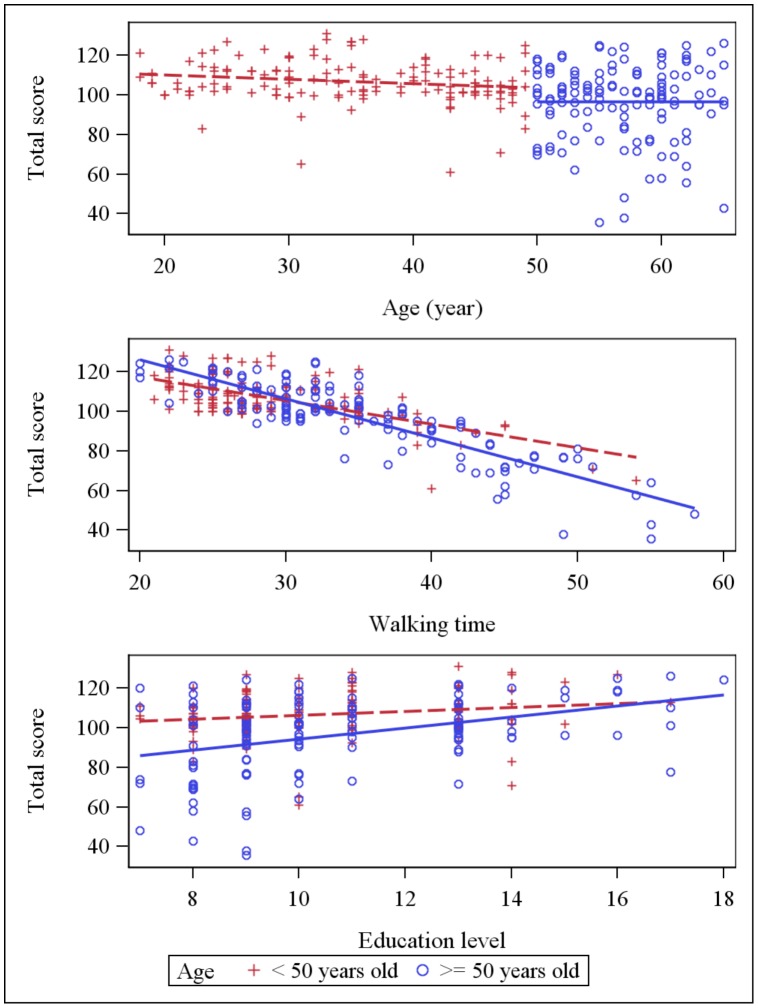
Scatter plot: Total psychometric score and explaining variables (age, education and walking time). Dot plots and linear regression for total psychometric score against the main explaining variables: age, education and walking time. Subjects <50 years old are represented by red crosses, subjects ≥ 50 years old are represented by blue circles.

Simple linear regression analyses showed that walking time was negatively correlated with all psychometric tests and TS showed the strongest correlation with walking time R = −0.817, p<0.0001 (Table 2). The dot plot of individual walking time and TS data revealed that the data were well fit by a linear regression curve (Figure 1a). Scatter plots for psychometric scores (each cognitive test and total psychometric score) against age, education and walking time are presented in Figures 2–6 for both age subsets.

**Table 2:** Simple regression - Relationship between cognitive test performance and walking time.

	Simple regression	
	Linear regression	
	equation (Y)	R squared
**MMSE global**	−0.279x+36.28	0.455
**Rey copy**	−0.411x+46.12	0.502
**Rey memory**	−0.564x+36.33	0.352
**Benton**	−0.447x+37.19	0.512
**Total psychometric score (whole)**	−1.701x+155.93	0.668
≤ 49 years old	−1.195x+141.28	0.413
≥ 50 years old	−1.976x+165.55	0.752

Simple linear regression with the cognitive tests scores as explained variables and walking time as explanatory variable.

In the step-wise multiple linear regression analysis, walking time was the first explanatory variable (among walking time, gender, height and weight) of the cognitive tests scores after adjustment for age and education (Table 3). The model showed that the unique contribution of walking time on cognitive scores was high whereas the other variables (gender, height and weight) had a low influence. Walking time strongly contributed to all psychometric tests and particularly to the TS (Table 3). These results were confirmed by the hierarchical multiple linear regression, with all potential confounders except walking time first forced into the model (age, education, gender, height and weight) and then walking time (Table 4).

**Table 3:** Step-wise multiple regression - Relationship between cognitive test performance and walking time.

	Step-wise multiple regression			
	Multiple R squared table			
	First step	Second step	Final model	
**Explanatory variables**	Age	Age	All	Unique
	Education^(a)^	Education	confounders^(c)^	contribution of
		Walking time^(b)^		walking time ^(d)^
**MMSE global**	0.084	0.463	0.484	0.379
**Rey copy**	0.121	0.506	0.518	0.385
**Rey memory**	0.102	0.367	0.376	0.265
**Benton**	0.191	0.527	0.537	0.336
**Total psychometric score (whole)**	0.184	0.680	0.692	0.496
≤ 49 years old	0.071	0.432	0.461	0.361
≥ 50 years old	0.146	0.759	0.789	0.613

Step-wise multiple linear regression with the cognitive tests scores as explained variables and walking time, age, gender, education level, height and weight as explanatory variables:

a) First step, age and education level are forced into the model,

b) Second step, walking time enters the model as first explanatory variable,

c) Then, the other confounders (gender, height, weight) were added one by one, R-squared for the final model is given.

d) Delta multiple R-squared between second step and first step, all p<0.0001.

**Table 4:** Hierarchical Multiple regression - Relationship between cognitive test performance and walking time.

	Hierarchical Multiple regression		
	Multiple R squared table		
**Explanatory variables**	^(a)^Age	^(b)^	^(c)^
	Education	+	Unique
	Gender	Walking time	contribution of
	Height		walking time
	Weight		
**MMSE global**	0.116	0.483	0.367
**Rey copy**	0.146	0.518	0.372
**Rey memory**	0.145	0.376	0.231
**Benton**	0.200	0.536	0.336
**Total psychometric score (whole)**	0.223	0.692	0.469
≤ 49 years old	0.079	0.465	0.386
≥ 50 years old	0.242	0.764	0.522

Hierarchical multiple linear regression with the cognitive tests scores as explained variables, age, education, gender, height and weight as explanatory variables:

a- First: age, gender, education level, height and weight were forced into the model

b- Then walking time was added to the model “a”

c- Unique contribution of the walking time (R-squared of model b – model a), all p<0.0001.

In order to check if this correlation was only due to the population having low cognitive performance, the correlation analysis was also performed in the 128 subjects with a total cognitive score higher than 104 (maximum TS score  =  132). The correlation coefficient between walking time and TS was lower in this subset but still significant (R = −0.218, p = 0.01).

### Relationship between TS and walking time in the 2 age subsets

Simple linear regression analyses showed that TS exhibited the strongest correlation with walking time in the subset of subjects aged 50 years old and over (R = −0.867; p<0.0001; Table 2). The dot plot of individual walking time and TS data from subjects aged 50 years old and over was well fit by a linear regression curve (Figure 1b). Table 3 shows results of the step-wise multiple linear regression analysis for the 2 age subsets. In both subsets, after adjustment for age and education, the unique contribution of walking time on TS was high compared to the other variables (gender, height and weight). The same results were obtained when performing a hierarchical multiple linear regression (Table 4).

Among the 128 subject having a total cognitive score higher than 104, 78 subjects were in the younger subset and 50 were in the older subset. Descriptive statistics for TS and walking time were similar in both subsets (details not shown) with mean values respectively for the younger and older subsets: TS = 113; 114 and walking time (in sec)  = 26; 28. A significant correlation between walking time and TS was found in the older subset (R = −0.393, p = 0.009) but not in the younger one (R = −0.192, p = 0.091).

## Discussion

To our knowledge, this study is the first to reveal a strong association between gait and cognition in a healthy middle-aged adult population because published papers in the field have mainly reported findings in elderly populations. Despite the use of different methodologies across studies, our findings in middle-aged adults align well with findings obtained in the elderly. Our findings are of interest for at least three reasons. First, although having normal global cognitive functioning, a substantial proportion of healthy adults exhibits a low performance in some aspects of visuospatial memory but without being obviously abnormal. Second, walking time was strongly correlated with the TS and differentially associated with individual tests. Compared to other factors known to influence gait and cognition (age, education, height or weight), walking time mainly contributed to cognitive performance, particularly to the TS. Third, considering the good explanatory rate of cognitive performances by walking time, GV can be considered as an adequate indicator of cognitive performance in a healthy adult population.

### Walking time assessment

In the absence of sophisticated technology measuring important variables such as rhythm, length and variability of step, walking speed was considered to be somehow the resultant of these variables and would implicitly indicate the quality of these different variables. The walking task used in our study wanted to be as close as possible to a natural fast walk. Our walking test included some motor and cognitive levels that despite their apparent simplicity made it a complex walk with several tasks. The order “walk, as fast as you can, without running, touch the wall, come back” puts the subject in a physical and mental challenge, requiring a certain degree of motivation. The turning phase of the task explored another aspect of locomotion speed and may resemble walking while avoiding obstacles. Our walking task explores the speed of psychomotor execution at lower limb with permanent cognitivo-motor adjustment. A recent study found that the turning was associated with specific cognitive demands such as information processing speed [22]. In order to efficiently manage his movements during the task, the subject needed to assess and memorize his environment but also spatially assess his own self. The walking task including the turning probably involved more cerebral structures, circuits and neurobiological processes than would do usual walk [23]. Most studies on gait explore usual walk, whereas fast walking could give more information. Normative data of walking speed in different age groups vary according to the task, the methods (e.g. computerized walkways, stopwatch, accelerometer, gyro) and the distances. Short distance walks are usually studied (as short as 3 meters) and measurements are often made using computerized walkways (total length of the mat varies from 3 meters to 10 meters) the beginning (acceleration phase) and the end of the walk (slowing down phase) are not recorded [5], [7], [22]. With this methodology, the walking distance is reduced and therefore requires several crossings of the mat in order to obtain sufficient number of gait cycles. It is difficult to compare the results of these studies to ours as the methodology is quite different; however we noticed that the mean walking speed in our two age subsets was slightly under the speed described by others [24]. This difference might be explained by the turning, acceleration and slowing down phases that we included in the walking time. In our study, the walking distance is quite long and raises the question on whether fast walking can be maintained over long distances. This point probably deserves further explorations.

### Cognitive assessment and performance

Hundreds of neurocognitive tests have been developed and different scoring methods have been described for a single test. Choosing the right test battery can be difficult knowing that various mental areas are entangled thus not obvious to individualize. In the present study we chose 3 widely known tests exploring a broad range of cognitive domains. They were also easy to administer and with a short duration.

Our global population had cognitive test scores within the normal range, indicating a normal cognitive functioning. It should however be noted that a substantial proportion of the subjects exhibited low performance in some aspects of visuospatial memory, few were obviously abnormal. As in any measurement, psychological test measures are not precise and have zones of uncertainty; thereby our ratio of low cognitive performers needs to be tempered. Normative data for the cognitive tests that we used describe several cut-off scores corresponding to different levels of performance, going from “very good performer” to “obviously altered” [18], [19]. In the present study, the superior normality cut-off scores were used in order to detect subtle cognitive alterations.

About half of the study population had a low cognitive performance. This raises the question whether our population can be considered as a representative sample of the healthy population. The healthy state of the subjects was carefully assessed with strict inclusion criteria and this is a strong point of our study. Moreover, as the population was composed of different socio-demographic categories and because the recruitment was not targeted, we believe that the study population was probably representative of the general healthy adult population. Having abnormal scores on some neuropsychological tests has already been reported in healthy adults and can affect about 20% of the subjects [25]. This finding is in itself epidemiologically significant and would need to be further explored. The comparison of the two age range subsets of subjects shows that low performance in some aspects of visuospatial memory can also take place in subjects <50 years old. To illustrate this point, low performance in the Rey memory test concerned a large proportion of younger subjects (approximately 69% of the subjects had a score under percentile 50) while for the other tests, the mean scores of younger subjects were typically in the normal range. These results are consistent with the hypothesis that an early onset of cognitive decline can be observed [12], [16], [26] although this view has been challenged [27].

A drop in the performance on all cognitive tests except the MMSE test (assessing overall cognitive functioning) was observed for subjects over 50 years old, leading to low mean scores especially for visual retention and discrimination of geometric form (Benton test). The above observations suggest that visuoconstructive abilities, visual retention and discrimination of geometric forms and overall cognitive functioning are relatively preserved until the approximate age of 50. The 50s and 60s may therefore be the approximate onset age for triggering poorer cognitive performance [28], [29]. The domain of spatial memory is broad and heterogeneous, and includes a wide range of processes and components [30]. As age increases, complex tasks requiring memory for spatial connection between several items and/or objects are less preserved than tasks requiring the ability to remember two or four distinctive localizations in space [31]. These considerations may explain the divergent results obtained in the Rey memory and Benton tests. Spatial cognition is crucial to everyday life; however few studies have examined the relationship between spatial cognition and neural changes with aging [32].

### Relationship between walking time and cognition

The relationship found between walking time and total cognitive performance was strong and potential confounding factors such as age, height, weight or education did not alter this relation. In all groups, walking time was the main explanatory variable of the total psychometric score. Our conclusion can be considered as robust since similar results were obtained when performing two different statistical approaches: walking time is the best explanatory variable of cognitive scores, particularly of TS, when the other confounding factors age, gender, education, height and weight are taken into account. Our findings suggest that GV could be an indicator of cognitive performance in healthy adults, particularly in the older subset. In spite of the great variety of neurocognitive assessment tools and gait tasks, literature confirms the existence of a link between gait speed and cognitive functions. Our results are in line with findings in elderly subjects and most importantly, indicate that the relationship between gait and cognition also exists in healthy middle-aged adults.

Interestingly, when cognitive performance was good, walking time was a weaker indicator of cognitive performance. This was probably due to a wider dispersion of the data. At this time, no clear explanation can be given to these findings but they show that the relationship between gait and cognition is the strongest when performances are not too high. Conversely, it would be interesting to investigate whether such correlation exists in severely demented patients could be an interesting research field. Visuospatial skills have an important effect on GV [33], [34], [35], [36] and are also very sensitive to aging [32]. Impairment of visuospatial skills is a prevalent early sign in the development of dementia [37], and abnormal gait patterns can predict the risk of future cognitive impairment [4], [8]. An interconnection between walking and mental functions, classically reported in clinical observations [38], [39], also coincides with structural and neuroimaging findings that establish common cortico-subcortical, temporo-limbic, hippocampal and cerebellar structures and networks [23], [33], [35], [40], [41], [42] for these activities. These common cerebral substrates could explain why a relation between gait and cognition can also exist in healthy adults, regardless of age. Because spatial performance tends to decline more rapidly than verbal performance with aging [14] and that all types of spatial memory become obviously altered in complex situations or tasks demands [32] it may be conceivable that dysfunction in these neural networks starts several years before the appearance of clinical signs [4], [43]. Additionally, a compensatory system, similar to that reported for semantic memory [44], may be present in people younger than 50 years old to maintain a rather stable mean cognitive performance and GV despite altered visuospatial memory. Indeed, compensational mechanisms in visuospatial memory are supported by neuroimaging data, where functional reorganizations appear with aging and recruit wider cerebral areas than seen in younger subjects [32]. To date, we cannot explain why in the good cognitive performers group, the link between gait and cognition is only present in older subjects and not in the younger ones. However, as similar total cognitive score and walking time were observed in both age subsets of good performers, analyzing brain functioning (e.g. using functional neuroimaging, electrophysiology) should give clues to understand the physiological bases of this finding. This interesting finding warrants more investigation in larger samples.

Though significant, the weak correlation between walking time and the Rey memory test suggests that visuospatial memory of many details or objects and their interconnection (Rey memory) is less important for a fast GV than a good memory for spatial locations of global geometric figures (Benton test) or objects. Visuospatial memory of many details could however be an important factor for a good mental imagery and navigation, which are complex components of spatial cognition but beyond the scope of this study. The total cognitive score had the best correlation with walking time. This result was not so surprising considering that an overall psychometric score allows the exploration of wider cognitive domains than an individual test. For example, the Benton test and the ROCFT explore different domains of spatial cognition. The Rey copy mainly explores perception and analysis strategies, organizational capacities and constructional approach when drawing the figure. This subtest does not involve visuospatial memory but mostly executive functions as those required when fast walking. The Rey memory, having the lowest correlation with walking time, explores spatial working memory of details and their interconnections without or little involvement of executive functions. The Benton test uses the spatial analysis skills of spatial memory, working memory and a certain type of executive function for the discrimination of pictures; its correlation with walking time was substantial. In summary, each test explores part of the spatial cognition whereas the overall cognitive performance covers several subdomains of spatial cognition.

The characteristics of our walking task could also explain this strong correlation with the total cognitive score, as before starting to walk, the subject had to record and memorize the instruction but also assess the characteristics of the environment and memorize them as a mental picture. During the walk, the subject had to continuously update his environmental information. Walking is a translocation of the body and involves many subdomains of spatial cognition such as spatial perception, mental imagery, spatial and working memory or executive functions [45].

A number of limitations should be emphasized. First, although the cognitive exploration used here covered global cognitive functioning and other cognitive factors, including visuospatial factors, a thorough neurocognitive assessment could be extended to include other cognitive tests and domains. Second, complementary types, conditions and characteristics of gait such as dual task, gait variability, step length and normal *versus* fast gait etc., could be analyzed to confirm or extend some of our findings [46]. Third, the cross-sectional nature of the design only gives information about walking time and cognition at a specific time point, and not their evolution with time or the predictability of gait speed on cognitive decline [47].

Collectively, our findings support the hypothesis that a significant proportion of apparently healthy adults without cognitive complaints have a low cognitive performance correlated with a longer walking time (i.e. lower walking speed), together with a dysfunction of certain aspects of visuospatial memory, and indicate that these low performances are more easily detectable from the age of 50. The grounds for such low performances are multiple, and their examination is beyond the scope of our study. However, this is an important finding that deserves further research. The administration of even simple cognitive screening tests is not easy and may even appear incongruous to a subject with no mental complaint. In contrast, GV is a rapid measurement that is easy to administer. GV can be considered as an adequate indicator of cognitive performance in an adult population. If our findings can be confirmed over shorter distances (e.g., 3–10 meters), GV could be routinely measured during a classical physical examination, and the collected data could indirectly inform the clinician about the cognitive status of healthy middle-aged adults who are without amnesic complaint but who are nevertheless at hidden risk for further cognitive decline. As stated in the introduction, the rationale for promoting such a test is that the earlier the detection – even prior to the onset of possible MCI – the better the probability of success for future interventions.
